# Attitudes toward harm reduction and abstinence-only approaches to alcohol misuse among Alaskan college students

**DOI:** 10.3402/ijch.v72i0.21143

**Published:** 2013-08-05

**Authors:** Monica C. Skewes, Vivian M. Gonzalez

**Affiliations:** 1 Department of Psychology, Center for Alaska Native Health Research, University of Alaska Fairbanks, Fairbanks, AK, USA; 2 Department of Psychology, University of Alaska Anchorage, Anchorage, AK, USA

**Keywords:** hazardous drinking, treatment strategies, emerging adults

## Abstract

**Background:**

Harm reduction is a public health approach that aims to guide hazardous drinkers to change unsafe drinking and minimize alcohol-related consequences without requiring abstinence. In contrast, abstinence-based interventions are designed for people with more severe alcohol problems and they aim to eliminate consequences via complete abstinence from alcohol. Current best practices for treating college student alcohol misuse involve harm reduction strategies, but no research has been conducted examining students’ perceptions of these strategies.

**Objective:**

Understanding attitudes is critical prior to the implementation of an intervention in a new setting, particularly when attitudes may serve as barriers to treatment enrolment and retention. For this reason, we sought to examine attitudes toward contrasting alcohol misuse interventions among college students in two large public universities in the circumpolar north.

**Design:**

A web-based survey was conducted with 461 students from two public universities in Alaska. Participants completed questionnaires assessing attitudes toward alcohol treatment, current drinking behavior, and demographic information.

**Results:**

Findings indicated that emerging adult (18–25 years old) students who would be targets of future interventions (hazardous drinkers) evidenced more positive attitudes toward harm reduction than abstinence-only approaches.

**Conclusion:**

This research provides support for the implementation of harm reduction intervention strategies for Alaskan college students who misuse alcohol. It is likely that harm reduction will be acceptable in this population.

Nearly half of U.S. college students report engaging in binge drinking (5+ drinks on one occasion for men or 4+ for women) within the past 2 weeks (1,2). It has been estimated that 31% of college students have symptoms consistent with a diagnosis of alcohol abuse and 6% with alcohol dependence (3). Heavy drinking in this population is associated with a number of serious consequences, including academic, health, legal, and social problems (4). Despite these consequences, alcohol misuse remains a common occurrence among college students and is a national public health concern (5).

Harm reduction (HR) is a public health approach that aims to guide problem drinkers to change unsafe drinking and minimize alcohol-related consequences without requiring abstinence (6,7). It aims to intervene before alcohol misuse develops into a more severe alcohol use disorder and, hence, is a form of secondary prevention. HR may include activities such as moderating alcohol consumption, engaging in protective behavioral strategies such as using a designated driver, changing one's thinking about alcohol use, learning and practicing drink refusal skills, and challenging alcohol-related social norms and expectancies. Abstinence-based interventions also are used to address problem drinking behavior but instead advocate complete avoidance of alcohol and alcohol-related cues to eliminate problems associated with excessive drinking. Abstinence is not incompatible with HR, but the decision regarding the goal of HR interventions (abstinence or controlled drinking) is left to the individual, and techniques meant to facilitate abstinence, such as the avoidance of drinking cues, are not emphasized. Abstinence-only (AO) and HR approaches both have been useful for improving health outcomes among people engaging in hazardous drinking; however, current best practices for treating college student alcohol misuse involve HR strategies (8).

Although few studies have examined attitudes toward HR among substance use disorder patients, findings suggest that clients’ beliefs about the need for abstinence affect preference for a moderation goal (9). Those who are younger (10), have less severe alcohol dependence (11), and greater social support for drinking (9) are more likely to select moderation than abstinence as a treatment goal. These characteristics describe college student drinkers; however, no published study to date has explored attitudes toward HR and AO interventions. The current study fills this gap in the literature by reporting on attitudes toward HR and AO treatment modalities in a sample of college students in the circumpolar north. Understanding attitudes toward different treatment approaches is important for improving the social validity and acceptability of interventions. For example, knowing how acceptable and appealing HR and AO strategies are to potential intervention participants can guide and inform future intervention efforts, and understanding factors that affect attitudes toward these treatment strategies can provide an evidence base for matching intervention approaches to client characteristics. This formative research constitutes a first step toward determining the type, components, and aspects of interventions that might be needed for Alaskan college students.

## Method

### Participants

Participants were 461 college student females (73.5%, n=339) and males (26.5%, n=122) attending large, open enrolment universities in Alaska. Students were surveyed from two universities, each providing approximately half the participants in the final sample (51.4 and 48.6%).

### Measures

#### Treatment Attitudes Scale (TAS)

At the time of this study, there was no published assessment instrument to examine attitudes toward HR and AO alcohol intervention techniques. Therefore, for the purposes of this study, the authors generated 46 items to assess participants’ attitudes toward the effectiveness of techniques that primarily are associated with either HR (e.g. “when you drink, try and space your drinks out”) or AO (e.g. “completely avoid places you used to drink”) interventions. Items representing the intervention strategies were rated for perceived effectiveness on a five-point scale from 1 (not at all likely to be effective) to 5 (very likely to be effective). Participants were provided the following directions: “There are many ways to manage problematic alcohol use. Imagine that you have an alcohol problem. Below are different types of things that you could do to try to change your problem. Rate how effective you think each strategy would be if you had an alcohol problem.”

Participants also completed a companion measure of self-efficacy for HR and AO intervention techniques. This companion self-efficacy measure consisted of the same 46 items that were rated for effectiveness, but the instructions and response options were different. Instead of asking how effective each strategy would be, the companion measure asked how confident participants were that they could successfully perform each strategy, rated on a five-point scale from 1 (not at all confident) to 5 (extremely confident).

Before using the TAS in this study, analyses were conducted to examine the psychometric properties of the instrument. An exploratory factor analysis using principal components analysis with equamax rotation resulted in a two-factor solution representing HR and AO techniques. Items with cross loadings (>0.30) or loadings below 0.50 were eliminated, resulting in 13 items per subscale. Loadings ranged from 0.56 to 0.78 on HR and 0.51 to 0.81 on AO. These subscales had high internal consistency with alpha coefficients of 0.93 for HR and 0.92 for AO. The self-efficacy companion measure also was subjected to a principal components analysis with equamax rotation, resulting in a two-factor solution representing HR and AO. The same items as those in the effectiveness subscales were retained, with few cross loadings evident. A total of five items with cross loading and one item with a loading below the previously used cut-off of 0.50 (0.47) were retained to allow direct comparison between the HR and AO scales. Loadings ranged from 0.61 to 0.74 on HR and 0.47 to 0.85 on AO. Self-efficacy subscale coefficient alphas were 0.93 for HR and 0.92 for AO.

#### Hazardous drinking

The Alcohol Use Disorder Identification Test–Consumption (AUDIT–C; 12) was used to categorize people as hazardous drinkers or non-hazardous drinkers. The AUDIT–C is comprised of three self-report items measuring: (a) frequency of drinking, rated from 0 (never) to 4 (four or more times per week); (b) drinks per drinking day, rated from 0 (1 or 2) to 4 (10 or more); and (c) frequency of having 6+ standard drinks on one occasion, rated from 0 (never) to 4 (four or more times per week). Items are summed to yield a total score, with higher scores indicating a greater likelihood of having an alcohol use disorder. Male participants who scored ≥4 and female participants who scored ≥3 were classified as hazardous drinkers (13).

#### Demographics

Participants were instructed to indicate their age, gender, and ethnicity. Regarding age, participants were classified as either emerging adults (18–25 years old) or adults over age 25. Research suggests that emerging adulthood is an especially risky time for developing alcohol problems (14), and traditionally college students are in this age range. Both universities sampled for this study have a higher proportion of non-traditional college students who are over the age of 25 than is typical; therefore, we explored potential age differences in attitudes.

### Procedure

The study protocol was approved by the institutional review boards of both universities where the study was conducted. Participants were recruited via a university web-based research portal and via in-class announcements and received extra course credit for their time and effort. Participants were given the link to the online survey and instructed to visit the web page to give electronic informed consent and complete the survey.

### Analyses

Separate 2×3 mixed between-within-subjects analyses of covariance were conducted on (a) perceived effectiveness and (b) self-efficacy for the intervention strategies. The within-subjects independent variable was intervention type (HR, AO) and the between-subjects independent variables were hazardous drinking (non-hazardous drinker/non-drinker, hazardous drinker) and stage of adulthood (emerging adult, adult over 25). Gender was entered as a covariate in all analyses. A main effect for intervention was examined first. Then we examined interactions between intervention and gender, intervention and stage of adulthood, and intervention and hazardous drinking. Finally, we examined a three-way interaction between intervention, stage of adulthood, and hazardous drinking. SPSS version 19 was used to analyze the data, and alpha was set at 0.05.

## Results

Six hundred thirty participants initiated the online survey. Participants with missing data (n=92), those with invariant responding (n=7), those who took less than 30 minutes to complete the survey (n=39), and those who took more than 4 hours (n=31) were eliminated. Analyses were conducted with the remaining 461 participants.

Participants’ mean age was 23.7 years old (*SD*=6.7), with a range from 18- to 54-years-old. The sample was 70.1% White/European American, 8.5% Alaska Native or American Indian, 7.2% Asian, 5.2% Hispanic/Latino, 4.8% African American, 2.6% mixed race/ethnicity, and 1.8% were other ethnic minorities or refused to answer. The majority of participants were full-time students (85.3%). The sample was 24.3% freshman, 23.4% sophomore, 22.3% junior, 27.8% senior, and 2.2% graduate students.

In this sample, 20.2% (n=93) reported not drinking and 79.1% (n=368) reported drinking at least once a month. A little over half of participants reported drinking six or more drinks on one occasion at least once a month (54.7%, n=252) and 42.1% (n=194) were hazardous drinkers based on AUDIT–C cut-off scores. Non-drinkers were classified as non-hazardous drinkers in the analyses. Means, standard deviations, and correlations among the study variables are presented in Table I.

**Table I:** Means, standard deviations, and intercorrelations of study variables

Variables	*M*	*SD*	1	2	3	4	5	6
1. Effectiveness–AO techniques	3.51	0.87	–					
2. Effectiveness–HR techniques	3.52	0.82	0.39***	–				
3. Self-efficacy–AO techniques	3.12	0.90	0.38***	0.14**	–			
4. Self-efficacy–HR techniques	3.67	0.81	0.17***	0.60***	0.46***	–		
5. Stage of adulthood	–	–	0.02	−0.20***	0.05	−0.10*	–	
6. Hazardous drinking	–	–	−0.15**	0.00	−0.32***	−0.02	−0.03	–
7. Gender	–	–	−0.13**	−0.11*	0.01	0.07	−0.01	−0.06

*N*=461. AO=abstinence-only, HR=harm reduction. Stage of adulthood was coded: emerging adult/18–25 years=0.26+ years=1. Gender was coded: men=1, women=0.

* p<0.05

** p<0.01

*** p<0.001

### Effectiveness

For the analysis of covariance (ANCOVA) examining perceived effectiveness, no significant main effect was found for intervention type, *F*(1,456)=1.68, p=0.195, η^2^=0.003. However, significant interactions were found between intervention type and both stage of adulthood, *F*(1,456)=13.28, p<0.001, η^2^=0.027, and hazardous drinking group, *F*(1,456)=17.12, p<0.001, η^2^=0.034. There also was a significant three-way interaction between intervention, hazardous drinking, and stage of adulthood, *F*(1,456)=8.47, p=0.004, η^2^=0.017. Gender did not significantly interact with intervention type, *F*(1,456)=0.32, p=0.57, η^2^=0.001.

To further examine these interaction effects, separate ANCOVAs examining the perceived effectiveness of intervention were conducted for hazardous drinking groups by stage of adulthood (see Table II). These analyses revealed that for non-hazardous drinking adults over 25 years of age, abstinence-only was perceived as much more effective than HR, *F*(164)=18.77, p<0.001, η^2^=0.226. In contrast, for emerging adult hazardous drinkers, HR was perceived as moderately more effective than AO, *F*(1,149)=6.14, p=0.014, η^2^=0.040. There were no significant differences in the perceived effectiveness of HR and AO for emerging adult non-hazardous drinkers, *F*(1,199)=0.54, p=0.462, η^2^=0.003, or for hazardous drinkers over the age of 25, *F*(1,456)=0.033, p=0.857, η^2^=0.001.

**Table II:** Effectiveness and self-efficacy for intervention techniques according to stage of adulthood and hazardous drinking

	Emerging adult (18–25)		Adult (26+)	
		
	Non-hazardous (n=201)	Hazardous drinker (n=151)	Non-hazardous (n=66)	Hazardous drinker (n=43)
				
Variables	Mean (SD)	Mean (SD)	Mean (SD)	Mean (SD)
Effectiveness				
AO techniques	3.59 (0.89)_a_	3.38 (0.80)_a_	3.71 (0.91)_a_	3.28 (0.87)_a_
HR techniques	3.65 (0.83)_a_	3.56 (0.71)_b_	3.11 (1.01)_b_	3.39 (0.62)_a_
Self-efficacy				
AO techniques	3.31 (0.84)_a_	2.81 (0.78)_a_	3.54 (0.99)_a_	2.65 (0.90)_a_
HR techniques	3.74 (0.85)_b_	3.66 (0.64)_b_	3.48 (1.05)_a_	3.60 (0.69)_b_

AO=abstinence-only, HR=harm reduction. For effectiveness and self-efficacy variables (separately), means in the same column with different subscripts are significantly different.

### Self-efficacy

For the ANCOVA examining self-efficacy, a large significant main effect was found for intervention type, *F*(1,456)=121.55, p<0.001, η^2^=0.187. Significant interactions were found between intervention type and both stage of adulthood, *F*(1,456)=4.69, p=0.031, η^2^=0.007, and hazardous drinking group, *F*(1,456)=56.78, p<0.001, η^2^=0.087. There also was a significant three-way interaction between intervention, hazardous drinking, and stage of adulthood, *F*(1,456)=9.96, p=0.002, η^2^=0.015. Gender did not significantly interact with intervention type, *F*(1,456)=1.88, p=0.171, η^2^=0.003.

To further examine these interaction effects, separate ANCOVAs examining self-efficacy for using the intervention techniques were conducted for hazardous drinking groups by stage of adulthood (see Table II). For non-hazardous drinkers over the age of 25, there was no significant difference in self-efficacy between HR and AO, *F*(1,64)=0.025, p=0.876, η^2^=000. However, for hazardous drinkers over 25, self-efficacy for HR techniques was significantly higher than for AO techniques with a large effect size, *F*(1,41)=31.04, p<0.001, η^2^=0.427. Self-efficacy for using HR techniques also was much greater than for AO techniques for emerging adults who were non-hazardous drinkers, *F*(1,199)=43.63, p<0.001, η^2^=0.179, as well as for those who were hazardous drinkers, *F*(1,149)=145.81, p<0.001, η^2^=0.492.

## Discussion

Significant differences in attitudes toward HR and AO were found based on drinking status and age group. Students in the older age group (26+) who were non-hazardous drinkers perceived AO to be more effective than HR; however, hazardous drinkers in this age group perceived both treatments as equally effective but reported significantly greater self-efficacy in their ability to perform HR strategies. Among emerging adults, non-hazardous drinkers also perceived the approaches to be equally effective and reported significantly greater self-efficacy for HR. However, emerging adult hazardous drinkers reported both greater perceived effectiveness and greater self-efficacy for HR than for AO intervention techniques.

These findings provide strong support for the acceptability of HR interventions for alcohol misuse among college students who are hazardous drinkers. Hazardous drinkers in both age groups had greater self-efficacy for using HR compared with AO techniques, and emerging adults also viewed HR as more effective. Alcohol interventions on college campuses target hazardous drinkers, and most college students are emerging adults (15). Therefore, the findings that emerging adult hazardous drinkers perceive HR as more effective and report greater self-efficacy for HR indicate that this type of intervention is more likely to be accepted by this at-risk group. It is interesting to note that, with the exception of older students who are not hazardous drinkers, students generally had greater self-efficacy for HR than AO, even when they did not perceive HR as more effective than AO. It makes sense that students in the alcohol-promoting college environment would feel greater confidence in their ability to moderate their drinking than to avoid alcohol and alcohol-related cues altogether. Because research shows that self-efficacy predicts success in alcohol treatment (16), it is noteworthy that self-efficacy was greater for HR for most students and for all hazardous drinkers. Because interventions will be designed for and targeted to hazardous drinkers, the greater self-efficacy for HR supports the use of HR approaches to treatment.

### Limitations and future directions

This research constituted a first step toward developing an alcohol misuse intervention that will be accepted by and effective for college students in Alaska. Instead of transporting existing manualized interventions from other parts of the world, the authors decided to engage in careful a priori research to determine which programs or aspects of programs might be needed in Alaska. To do this, we first needed to understand the attitudes of students who may be targets of future interventions. We gained valuable information about strategies that students think would be effective for reducing alcohol misuse and problems and that they also feel confident they could perform. This research suggests that HR would be a better approach than AO for addressing alcohol misuse among Alaskan college students.

A limitation of the current research is the overrepresentation of female college students and underrepresentation of Alaska Native college students compared to the overall population of Alaskan college students. Ongoing research is being conducted to explore attitudes solely among Alaska Native students who drink.

It also is important to note that attitudes do not necessarily predict outcome. Having positive attitudes toward HR does not ensure that an HR intervention will be effective. However, having negative attitudes toward HR would be a significant barrier to the successful enrolment and retention of participants in an HR intervention. Therefore, positive attitudes are necessary, but not sufficient, for intervention to be successful. Future research will be conducted to qualitatively explore students’ attitudes toward alcohol misuse interventions, with special attention paid to the perceptions of Alaska Native students who may have culturally distinct experiences with and perceptions of alcohol. Ultimately, an HR intervention that is acceptable and appropriate for college students in Alaska will be implemented and evaluated.
